# Role of Clinical Presentations and Routine CSF Analysis in the Rapid Diagnosis of Acute Bacterial Meningitis in Cases of Negative Gram Stained Smears

**DOI:** 10.1155/2014/213762

**Published:** 2014-04-03

**Authors:** Rabab Fouad, Marwa Khairy, Waleed Fathalah, Taha Gad, Badawy El-Kholy, Ayman Yosry

**Affiliations:** ^1^Endemic Medicine Department and Hepatology Unit, Faculty of Medicine, Cairo University, Cairo 11562, Egypt; ^2^Shebin El-Kom Fever Hospital, Egyptian Ministry of Health, Menoufia 12489, Egypt; ^3^Clinical Pathology Department, Faculty of Medicine, Cairo University, Cairo 11562, Egypt

## Abstract

*Background and Aim.* Bacterial meningitis is a lethal, disabling endemic disease needing prompt antibiotic management. Gram stained smears is rapid accurate method for diagnosis of bacterial meningitis. In cases of negative gram stained smears diagnosis is delayed till culture results. We aim to assess the role of clinical presentations and routine CSF analysis in the cost-effective rapid diagnosis of negative gram stained smears bacterial meningitis. *Methods.* Cross sectional study including 623 acute meningitis patients divided into two groups: bacterial meningitis and nonbacterial meningitis groups. The clinical presentations, systemic inflammatory parameters, and CSF analysis were evaluated and compared in both groups. *Results.* Altered conscious level, localizing neurological signs, Kernig's and Brudzinski's signs together with peripheral leucocytosis (>10.000/mm^3^), high CRP (>6) together with high CSF protein (>50 gl/dL), CSF neutrophilic count (≥50% of total CSF leucocytic count), and low CSF glucose level (<45 gm/dL) and CSF/serum glucose ≤0.6 were significantly diagnostic in bacterial meningitis patients. From the significant CSF analysis variables CSF protein carried the higher accuracy of diagnosis 78% with sensitivity 88% and specificity 72%. *Conclusions.* High CSF protein (>50 mg/dL) together with plasma inflammatory markers and CSF cytochemical parameters can diagnose bacterial meningitis in gram stain negative smear till culture results.

## 1. Introduction 

Acute bacterial meningitis is a major cause of death and disability worldwide. It affects over one million people yearly, with higher incidence among developing countries and in specific geographic areas [1].

Meningitisis an endemic disease in Egypt;* S. pneumonia* meningitis is currently the leading cause of meningitis in Egypt and has the highest mortality rates among meningitis cases especially in patients less than one year of age [2, 3].

Acute meningitis is caused by a variety of infectious agents. The most serious form is caused by pyogenic bacteria, such as* S. pneumoniae, N. meningitidis,* and* H. influenzae* [4]. Viruses are the most common cause of aseptic meningitis, primarily enteroviruses, together with numerous nonviral and noninfectious etiologies [5].

Differentiating bacterial from nonbacterial meningitis is very important in deciding treatment. Bacterial meningitis is a life-threatening neurological condition and needs prompt parenteral antibiotics, compared to viral and aseptic meningitis which carries relatively better outcome [6]. Delay in the start of proper therapy introduces the potential for increased morbidity and mortality, if the patient does indeed have acute bacterial meningitis [5].

CSF culture is highly specific but lacks sensitivity, especially when antimicrobials have been given as well as the time needed till results appear [7]. Someinvestigators document bacterial meningitis only in patients with positive CSF culture and/or positive latex agglutination test on CSF or positive blood culture with concomitant CSF pleocytosis [8, 9].

Gram stain smears of the CSF permits a rapid, accurate method of diagnosis of bacterial meningitis in 60%–90% of patients; the percentage correlates with the CSF concentration of bacteria [10]. In case of negative gram stained smear the differentiation between bacterial and nonbacterial meningitis is needed with other tools [11].

Identification of the causative agent by Gram staining unfortunately shows low rate and variability in sensitivity [12]. The yield of bacteria detection depends on several factors as the number of organisms present, prior use of antibiotics, and technique used for smear preparation [3, 13].

The classic CSF abnormalities in bacterial meningitis are a polymorphonuclear leukocytosis, decreased glucose concentration, and increased protein concentration. In viral meningitis, the classic CSF abnormalities are a lymphocytic pleocytosis, a normal glucose concentration, and a normal or slightly elevated protein concentration [14].

In sterile CSF after antibiotic intake in case bacterial meningitis white cells found in CSF are primarily polymorphs, meningitis is bacterial in origin, which may persist throughout the illness [15, 16].

Additional diagnostic tests are necessary to distinguish between bacterial and viral meningitis. The peripheral WBC count, CRP, and ESR are usually elevated in patients with bacterial meningitis [17].

## 2. Objectives

The aim of our study is to assess the role of clinical presentations, serum inflammatory markers including CRP, and routine CSF analysis in the rapid diagnosis of acute bacterial meningitis in cases of negative gram stained smears to reach a cost-effective diagnostic approach based on routine diagnostic labs.

## 3. Material and Methods

### 3.1. Selection of Patients

Three-year period (2009–2012) prospective cross-sectional study was done including 623 patients diagnosed as acute meningitis presented to Shebin El-Kom Fever Hospital, a tertiary care center specialized in endemic diseases and infectious diseases especially meningitis and encephalitis.

Patients were subjected to thorough history taking and clinical examination with special emphasis on symptoms of meningeal irritation: fever, headache, vomiting, photophobia, and irritability. Signs of meningeal irritation as neck rigidity, Kernig sign, Brudzinski sign, altered conscious level, seizures, focal neurological signs, skin rash were assessed. In infants symptoms of weak suckling and high pitched crying and bulging anterior fontanel were reviewed in addition. The clinical assessment was conducted by two specialized clinicians together in the same setting of the diagnosis.

Special concern was conducted on the associated infections and previous antibiotic intake and antecedent illness as pneumonia, otitis media, sinusitis, urinary tract infection, and diarrhea or previous surgical intervention.

Laboratory tests included complete blood count (CBC), C-reactive protein (CRP), and serum blood glucose. CSF analysis was done including total white cells count (neutrophils or lymphocytes), protein and glucose level, and CSF/serum glucose. CSF was subjected to gram stain and cultures which were inoculated onto chocolate, blood, and MacConkey agars. Ziehl Neelsen stain for mycobacteria tuberculosis and India ink preparation were done when tuberculosis meningitis and cryptococcal meningitis were clinically suspected, respectively. The laboratory tests were all done by expert clinical pathologist and equipments were calibrated to overcome bias.

### 3.2. Patients' Classification

Total of 623 patients studied there were classified into two groups:

1. Group I (*n* = 457 patients): bacterial meningitis with positive CSF culture or positive blood culture with concurrent meningitis,
2. Group II (*n* = 166 patients): nonbacterial meningitis with CSF and negative blood cultures.

### 3.3. Patients Consent

Informed written consent from each patient and local ethical committee approval were obtained before starting the data collection. With respect to patients' confidentiality, patients were represented in the study by code numbers. All personal data was concealed. The study protocol conformed to the ethical guidelines of the 1975 Declaration of Helsinki as reflected in a prior approval by the institution's human research committee.

### 3.4. Statistical Analysis

Data were collected and statistically analyzed using SPSS version 11 statistical package. Comparison of qualitative data was performed with chi-square (*χ* ^2^) test. Multivariate backwards stepwise binary logistic regression analysis with bacterial meningitis—as the dependent factor—was done. The validity of screening tests was measured and expressed as sensitivity, specificity, accuracy, positive predictive value, and negative predictive value (in comparison to diagnostic tests). *P* value <0.05 was considered significant.

Spearman correlation coefficient test was used for correlation between nonparametric quantitative data. Also, Mann-Whitney *U* test was used for comparison between nonparametric quantitative data between two groups.

## 4. Results

Among the studied 623 patients, bacterial meningitis represented 73.3% (457 patients) compared to 26.7% (166 patients) nonbacterial meningitis of the studied population. Bacterial meningitis carried a higher mortality rate 20.6% than nonbacterial meningitis being only 3.6%.

Among the bacterial meningitis patients, the isolated organisms on the CSF bacterial cultures (chocolate, blood, and MacConkey agar)* S. pneumoniae* was the most frequently isolated (52%) while* N. meningitidis* in 22.2% and* H. influenzae* in 14.8%.

### 4.1. Demographic Data of the Studied Patients

Patients with either bacterial or nonbacterial meningitis obeyed the same demographic features; that is, patients were distributed in all age groups, with low rates of occurrence in the extremes of age (the neonates and above 60 years). Men were affected more significantly than female patients as presented in Table 1.

### 4.2. Clinical Presentations of the Studied Patients

Antecedent illnesses (i.e., diseases diagnosed at the time or shortly before the diagnosis of meningitis) were recorded in 34% of the total patients with pneumonia recorded in 18.8% of the cases. Diarrhea, otitis media, and sinusitis were reported to a lesser degree. All cases of recurrent meningitis were due to posttraumatic CSF leak and mainly of bacterial meningitis origin (22 out of 24 patients). A significant proportion of meningitis patients (55.2%) reported a positive history of antibiotic intake in the few days (up to 72 hours) before admission to the hospital (Table 2).

Clinical presentations (e.g., fever, vomiting, and blurring of vision) were of little assistance in differentiating bacterial from nonbacterial meningitis, while signs of meningeal irritation as Kernig's sign, Brudzinski's sign, and altered conscious level and localizing neurological signs were found to be significantly higher in bacterial than nonbacterial meningitis group as shown in Table 3.

### 4.3. Diagnosis of Meningitis in the Studied Patients

The plasma inflammatory markers showed highly significant difference between both groups (*P* value <0.01). Leucocytosis (>10,000/mm^3^) was encountered in bacterial meningitis in 47.9% of patients, while only in 24.1% of patients with nonbacterial meningitis. Positive CRP result (≥6) was significantly higher in patients with bacterial (47.9%) than nonbacterial meningitis (15.7%) as shown in Table 4.

The CSF analysis of the studied patients also showed significant difference between the two groups of patients. Elevated CSF protein (>50 mg/dL) was present in 87.4% of patients with bacterial meningitis versus 47.6% of patients with nonbacterial meningitis. Decreased CSF glucose values (<45 mg/dL) were found in 46.8% and 15.7% of patients with bacterial and nonbacterial meningitis, respectively.

In patients with bacterial meningitis, 67.4% had a CSF leukocyte count in the range of >100–1,000 cell/mm^3^ and 32.6% had a leukocyte count >1,000 cell/mm^3^. Patients with bacterial meningitis had a predominantly neutrophilic CSF, that is, neutrophil percentage >50% (69.4%). On the other hand, patients with nonbacterial meningitis had a predominantly lymphocytic CSF in 76.5% of cases as in Table 5.

CSF/serum glucose ratios were calculated; 90.6% and 64.5% of patients with bacterial and nonbacterial meningitis were found to have a decreased ratio (CSF/serum glucose <0.6), respectively.

The multivariate binary logistic regression analysis was performed for the significant variables including peripheral leucocytosis, high CRP, and CSF analysis. Validation of the serum inflammatory parameters and the CSF analysis, sensitivity, specificity, accuracy, positive predictive value (PPV), and negative predictive value (NPV) were calculated in Table 6. Three parameters, CSF protein, neutrophil count, and CRP, appeared to have good predictive value in bacterial meningitis. On the other hand CSF glucose and peripheral blood leukocytosis appeared to be less efficient in the diagnosis. ROC curve was plotted with CSF protein having the best performing curve for diagnosing of bacterial meningitis with sensitivity of 88%, specificity of 72%, accuracy of 78%, positive predictive value of 84%, and negative predictive value of 60%.

## 5. Discussion 

Meningitis is an endemic disease in Egypt, with a higher reported incidence of bacterial meningitis ranging from 47% to 68% [2, 18, 19]. Bacterial meningitis can be lethal if not diagnosed or treated at an early stage. Usually Viral meningitis in immunocomptent and aseptic meningitis carries a good prognosis and get cured within one or two weeks without any treatment [20, 21].

It is evident that the clinical differentiation between bacterial and aseptic meningitis is challenging. Rapid diagnosis and treatment of acute community-acquired bacterial meningitis reduces mortality and neurological sequelae but can be delayed by atypical presentation, assessment of lumbar puncture safety, and poor sensitivity of standard diagnostic microbiology [22].

In endemic areas for bacterial meningitis as Egypt, such a serious disease with poor outcome in a relatively low resources setting and in an easy and rapid noncomplicating, cost affordable way of diagnosis based on routine diagnostic labs is important.

The aim of our study is to assess the role of clinical presentations, serum inflammatory markers including CRP, and routine CSF analysis in the rapid diagnosis of acute bacterial meningitis in cases of negative gram stained smears to start rapid treatment as early as possible without waiting culture results available in suspected cases of bacterial meningitis to overcome the lethal complications. Our study focused on the validation of clinical and routine diagnostic tests in bacterial meningitis and detection of the most discriminating factors with respect to nonbacterial meningitis.

In the current study, bacterial meningitis represents 73.3% of the studied population and nonbacterial meningitis accounted for the remaining 26.7%. The ratio of bacterial to aseptic meningitis cases differed between several studies [20, 23]. This difference can be attributed to differences in the place and time of studies and implementation of anticapsular vaccines [24].

In our study, meningitis in the preschool children represented 39.3% of the studied population. Other studies documented a higher prevalence up to 60–75% [25, 26]. The decline of rate of occurrence may be explained by the availability of effective vaccines against common pathogens (e.g., capsulated organisms) [27, 28].

The low incidence of meningitis in the extremes of ages does not allow the conclusion that younger/older patients are indeed less affected by bacterial meningitis; it may also mean that the group of patients with bacterial meningitis at the extremes of age are underrepresented in this study.

The dominating causative agent for bacterial meningitis has changed over the years in Egypt [29].* S. pneumoniae* represents 52% of the isolated organisms in our patients with antecedent pneumonia in 17.3% of bacterial meningitis cases. Pneumococcal meningitis is currently the leading cause of meningitis in Egyptdue to decrease in the incidence of meningococcal diseasereflecting the increased use of polysaccharide meningococcal vaccines. Similar pneumococcal predominance especially serotype 1 was recently noted at the African meningitis belt.

In our study, the triad of meningeal inflammation, that is fever, headache, and neck rigidity, was found in 87.5%, 80.5%, and 69.8%, respectively, with no significant difference between the two groups. Similar rates were reported by several investigators [3, 28, 30]. Kernig's and Brudzinski's signs were statistically significant in our bacterial meningitis patients. However, previous study noted that Brudzinski's sign and nuchal rigidity did not accurately discriminate between patients with meningitis, even bacterial meningitis, and patients without meningitis [31].

Altered level of consciousness and localizing neurologic signs were more significant in bacterial (44.9% and 5.5%, resp.) than nonbacterial meningitis patients (27.7% and 1.8%, resp.). These neurologic manifestations are related to the severity of the disease and the time interval before arrival to the hospital [23]. As previously reported, focal neurologic deficits and seizure activity do not commonly occur in aseptic meningitis [14].

Although around half of the studied patients (55%) were preexposed to antibiotics, this may not affect the diagnosis of bacterial meningitis as the duration of use of antibiotics was within 72 hours of the diagnosis. The leucocytic count and inflammatory markers of CSF remain positive even in sterile CSF after the use of antibiotics [32].

Considerable peripheral leucocytosis (>10.000/mm^3^) and raised CRP level (>6) was significantly higher in our bacterial meningitis group in 52.1% and 74.4% of patients, respectively. It is reported that plasma inflammatory markers such as peripheral blood leukocyte count and CRP can be very useful in discriminating between bacterial and nonbacterial meningitis [6, 28].

Meta-analysis from 35 studies proposed to use CRP as an additional tool for discriminating bacterial meningitis from viral meningitis, without having evaluated its independent contribution relative to other parameters such as white blood cell count, CSF white cell count, protein, or glucose [17].

Our bacterial meningitis patients showed significant CSF leucocytosis with neutrophilic predominance (69%) and high CSF protein (>50 mg/dL) (87.5%) compared to the nonbacterial meningitis group, 23.5% and 47.7%, respectively. In bacterial meningitis, CSF leukocyte count <1,000/uL may be found early in the disease, in partially treated bacterial meningitis, in overwhelming bacterial meningitis, and in immune-suppressed and leucopenic patients [16–18].

CSF glucose concentration is decreased <45 mg/dL in 46.8% of patients with bacterial meningitis and significantly above 45 mg/dL in 84.3% of the aseptic meningitis patients. CSF glucose is typically normal in aseptic meningitis, although it may be decreased in cases due to enteroviruses, HSV-2, and VZV meningitis [14].

Previous study showed that CSF to serum glucose of ≤0.4 was 80% sensitive and 98% specific for the diagnosis of bacterial meningitis in children below 2 months and at a value below or equal to 0.6 in the neonates [16]. In our study, 90.6% of the bacterial meningitis patients CSF/glucose level was <0.6.

Validation of the significant parameters in the CSF analysis showed that CSF protein concentration was of the highest sensitivity, specificity, and accuracy in diagnosis of bacterial meningitis in our study. Many studies reported the same finding with variation in the mean values between bacterial and aseptic meningitis patients [13, 28].

We conclude that for rapid diagnosis of bacterial meningitis in cases of negative gram stained smears the combination of high CSF protein content (>50 mg/dL) together with the signs of meningeal irritation, localizing signs, and cytochemical CSF analyses can expect bacterial meningitis in these cases till culture results appearance. The classic biological markers in blood in the form of high CRP and peripheral leucocytosis can increase the sensitivity of diagnosis without adding high cost compared to CSF culture and smears which are of low diagnostic yield.

PCR and agglutination tests were not used in the study which is considered as limitation of the study. However, the PCR is costly and the study is aiming to find a relatively cheap and accurate method of diagnosis in a set of large number of patients in an endemic area of the disease. The rapid tests in previous studies, although having good sensitivity, lack specificity, were not conclusive, and did not show a high sensitivity or specificity [6]. Nevertheless, the routine use of latex agglutination for the etiologic diagnosis of bacterial meningitis has recently been questioned [16].

We could not conclude that the negative parameters are not a rationale for exclusion of diagnosis of bacterial meningitis and it would be hard to justify not treating patients in the first hours of diagnosis with antibiotics or to suggest a nonbacterial etiology. Further studies are needed to reach this conclusion.

## Figures and Tables

**Table 1:** Demographic data of bacterial and nonbacterial meningitis in the studied population.

Demographic data	Group I (*n* = 457)		Group II (*n* = 166)		*P* value
	Number	%	Number	%	
Age					0.07
0-1 month	7*	1.5*	0^†^	0.0^†^	
>1 month–6 years	172	37.6	66	39.7	
>6–18 years	74	16.2	24	14.4	
>18–60 years	172	37.6	62	37.3	
>60 years	32*	7.0*	14^†^	8.4^†^	
*P*	<0.01*		<0.01^†^		
Sex					
Male	279	61.00	95	57.2	0.08
Female	179	39.00	71	42.8	
*P*	**<0.01**		**<0.01**		

*P value significant with these values ^†^ *P* value significant with these values.

**Table 2:** Underlying and associated conditions in bacterial and nonbacterial meningitis groups in the studied population.

Clinical manifestations	Group I (*N* = 457)		Group II (*N* = 166)		Total (*N* = 623)		*P*
	Number	%	Number	%	Number	%	
Fever	401	87.8	144	86.7	545	87.5	0.8
Headache	370	81.1	131	78.9	360	80.5	0.4
Vomiting	222	48.6	71	42.8	293	47	0.9
Irritability	134	29.3	43	25.9	177	28.4	0.3
Photophobia	42	9.2	15	9.00	57	9.1	0.5
Neck rigidity	328	71.8	107	64.5	3.09	69.8	0.5
Kernig's sign	187	40.9	54	32.5	241	38.7	**0.03**
Brudzinski's sign	193	42.2	52	31.3	245	39.3	**<0.01**
Skin rash	20	4.4	3	1.8	23	3.7	0.08
Altered conscious level	205	44.9	46	27.7	251	40.2	**<0.01**
Seizures	107	23.4	38	22.9	145	23.2	0.07
Localizing signs	25	5.5	3	1.8	28	4.5	**<0.05**
Anterior fontanel bulge	57/123	47.1	20/46	43.5	77/169	45.5	0.5
Abnormal crying	54/123	36.6	16/46	34.8	70/169	41.4	0.3
Weak suckling	43/123	35.00	16/46	34.8	59/169	35	0.5

**Table 3:** Clinical manifestations of bacterial and aseptic meningitis in the studied population.

Condition	Group I (*N* = 457)		Group II (*N* = 166)		Total (*N* = 623)		*P*
	Number	%	Number	%	Number	%	
Pneumonia	79	17.3*	38	22.9*	117	18.8*	
Recurrent meningitis	22	4.8	2	1.2	24	3.9	
Diarrhea	12	2.6	12	7.2	24	3.8	
Otitis media	19	4.2	2	1.2	21	3.4	
Sinusitis	15	3.3	3	1.8	18	2.9	
Urinary tract infection	0	0	6	3.6	6	1.0	
Cirrhosis	3	0.7	0	0	3	0.5	
Spinal anesthesia	0	0	1	0.6	1	0.2	

Total	150	33	64	38	214	34	<0.01*

Antibiotic intake							
Positive	275	60.2	69	41.6	344	55.2	**<0.01**
Negative	182	39.8	97	58.4	279	44.8	

*P value significant with these values.

**Table 4:** Plasma inflammatory markers inbacterial and nonbacterial meningitis groups in the studied population.

Inflammatory markers	Group I (*n* = 457)		Group II (*n* = 166)		*P* value
	Number	%	Number	%	
WBCs (/mm^3^)					
4.000–10.000 (mean ± SD)	10877.02 ± 5113.76		8093.37 ± 4488.99		<0.01
≤10.000	238	52.1	126	75.9	<0.01
>10.000	219	47.9	40	24.1	
CRP (mg/L)					
Normal (<6) (mean ± SD)	27.03 ± 28.07		4.24 ± 0.11		0.01
Positive (>6)	340	74.4	26	15.7	<0.01
Negative (<6)	117	25.6	140	84.3	

WBCs: white blood cells; CRP: C-reactive protein.

**Table 5:** CSF parametersmarkers inbacterial and aseptic meningitis groups in the studied population.

CSF parameters	Group I (*n* = 457)		Group II (*n* = 166)		*P*
	Number	%	Number	%	
Protein (mg/dL)					
Mean ± SD	135.89 ± 86.98		56.66 ± 24.53		**0.01**
<50	57	12.5	87	52.4	**<0.01**
>50	400	87.5	79	47.6	
Glucose (mg/dL)					
Mean ± SD	48.30 ± 28.78		63.96 ± 28.50		**0.01**
>45	243	53.2	140	84.3	**<0.01**
<45	214	46.8	26	15.7	
CSF/serum glucose					
>0.6	43	9.4	59	35.5	**<0.01**
≤0.6	414	90.6	107	64.5	
WBCs (total/mm^3^)					
Mean ± SD	3484.65 ± 10186.54		66.80 ± 23.45		**0.01**
≤100	0	0	166	100	**<0.01**
>100–1000	308	67.4	0	0	
>1000	149	32.6	0	0	
Neutrophil %					
Mean ± SD	61.65 ± 26.69		29.45 ± 27.65		**0.01**
>50	317	69.4	39	23.5	**<0.01**
≤50	140	30.6	127	76.5	

**Table 6:** Validation of CSF and blood parameters in detecting bacterial meningitis.

Parameters	Sensitivity (%)	Specificity (%)	Accuracy (%)	PPV (%)	NPV (%)
CSF protein	**88**	**52**	**78**	**84**	**60**
CSF glucose	47	84	57	89	37
CSF neutrophil	69	77	71	89	48
Serum CRP	74	84	77	93	54
Peripheral WBC	48	76	55	85	35

PPV: positive predictive value; NPV: negative predictive value.
